# Immunolocalization of aromatase and estrogen and androgen receptors in the gonads and mesonephros of *Podocnemis expansa* during the first year of life

**DOI:** 10.1590/1984-3143-AR2025-0050

**Published:** 2026-02-09

**Authors:** Maria Fabiele Silva Oliveira, Layla Ianca Queiroz Rocha, Lucas Castanhola Dias, André de Macedo Medeiros, Moacir Franco de Oliveira, Carlos Eduardo Bezerra de Moura, Marcela dos Santos Magalhães

**Affiliations:** 1 Programa de Pós-graduação em Biologia de Água Doce e Pesca Interior, Instituto Nacional de Pesquisas da Amazônia, Manaus, AM, Brasil; 2 Programa de Pós-graduação em Ciência Animal, Universidade Federal Rural do Semi-Árido, Mossoró, RN, Brasil; 3 Laboratório Temático de Microscopia Eletrônica e Nanotecnologia, Instituto Nacional de Pesquisas da Amazônia, Manaus, AM, Brasil; 4 Departamento de Ciências Animais, Universidade Federal Rural do Semi-Árido, Mossoró, RN, Brasil; 5 Departamento de Morfologia, Universidade Federal do Amazonas, Manaus, AM, Brasil

**Keywords:** morphophysiology, Amazon turtle, steroid hormones

## Abstract

The endocrine regulation of testicular and ovarian development during early life in chelonians remains poorly understood, despite major morphophysiological changes occurring in this period. This study investigated the immunolocalization of estrogen (ER1 and ER2), androgen (AR), and aromatase (AROM) in the gonads and mesonephros of *Podocnemis expansa* during the first year after hatching (n = 5; males: 1, 3, 12 months; females: 2, 4 months). After euthanasia, gonads and mesonephros were collected, fixed in 10% buffered formaldehyde, and processed for immunohistochemistry. The intensity of immunoreactions varied according to age and sex, but not with tissue distribution. AROM, AR, ER1, and ER2 were detected in both gonads and mesonephros, confirming the persistence of steroidogenic and steroid-responsive activity after hatching. In males, AROM showed weak to moderate labeling in germ and interstitial cells of seminiferous tubules, whereas ER2 expression predominated in spermatogonia and interstitial endocrine cells. In females, AROM and AR were moderately expressed in the germinal epithelium and oogonia at two months, decreasing at four months, while ER2 persisted in follicles and oogonia. These findings suggest a dynamic endocrine environment influencing posthatch gonadal maturation, with the mesonephros acting as a transient extragonadal source of steroids. Despite the small, sex-unbalanced sample (due to lack of dimorphism), the results provide valuable baseline information and support future quantitative and functional studies on the reproductive endocrinology of *P. expansa* and other chelonians with temperature-dependent sex determination.

## Introduction

The Amazon turtle, *Podocnemis expansa*, is the largest freshwater turtle in South America and has great ecological, economic and cultural relevance in the Amazon region (Hildebrand et al., 1997; Rueda-Almonacid et al., 2007; Vogt, 2008). Although its conservation status is classified as low risk, the species is heavily dependent on protection and management strategies, especially in breeding areas, both inside and outside protected areas (Rhodin et al., 2021). Illegal egg collection, habitat loss and climate change pose significant threats to the maintenance of their natural populations, making it essential to increase knowledge about their reproductive biology to support effective conservation actions (Souza and Vogt, 1994; Marcovaldi et al., 2011).

A fundamental aspect of the reproductive biology of *P. expansa* is incubation temperature-dependent sex determination (TSD), a mechanism widely documented in several species of turtles (Yntema, 1976; Bull and Vogt, 1979; Bull, 1980; Janzen and Paukstis, 1991; Valenzuela, 2001). In *P. expansa*, incubation of eggs at low temperatures favors the development of males, whereas relatively high temperatures result in a relatively high proportion of females (Valenzuela, 2001). This pattern of sex determination, together with hormonal factors, regulates the differentiation and maturation of the reproductive system of the species, and steroid hormones, such as androgens and estrogens, are responsible for playing an essential role in gonadal differentiation and functionality and in the formation of sperm pathways and oviducts (Morais et al., 2012; Gradela et al., 2020).

These hormones act through specific receptors, such as estrogen alfa (ERα, also known as ER1) and estrogen beta (ERβ, also known as ER2) and androgen (AR) receptors, which regulate processes such as gametogenesis, the cellular organization of the seminiferous tubules and germ cell development, and the development of follicles ovaries and oocytes (Delbès et al., 2006; Albrecht et al., 2009; Moore et al., 2010). Additionally, aromatase, the enzyme responsible for the conversion of androgens into estrogens, is fundamental in the regulation of the hormonal environment during sexual development in reptiles (Pieau 1996; Pieau and Dorizzi 2004; Matsumoto et al., 2013).

The mesonephros, an embryonic structure derived from the urogenital fold, also play an important role in initial hormonal synthesis and gonadal differentiation (Kardong, 2010; Macêdo et al., 2022). Reports indicate the presence of steroid hormone receptors and aromatase in the mesonephros of turtles during embryonic development (Magalhães, 2017; Macêdo et al., 2022). However, little is known about the dynamics of the expression of these components during posthatch gonadal maturation, as well as their possible implications for the future reproductive functionality of these animals.

The lack of information on the immunolocalization of estrogen (ER1 and ER2), androgen (AR) and aromatase (AROM) receptors in the gonads and mesonephros of *P. expansa* offspring in the first year of life represents a significant gap in knowledge about the reproductive development of this species. Understanding these mechanisms is essential not only to advance the morphophysiological knowledge of the species but also to support conservation and reproductive management strategies, especially in the face of environmental pressures, such as exposure to xenoestrogens and climate change (Mendonça et al., 2016).

Therefore, the present study aims to investigate the expression and distribution of steroid hormone receptors and aromatase during gonadal and mesonephros maturation in the posthatch period of *P. expansa*, contributing to the improvement of the knowledge of reproductive biology and providing insights for models applicable to other chelonian species with TSD.

## Methods

### Acquisition of samples

The hatchlings were collected at the Center for Research and Preservation of Aquatic Mammals and Chelonians, Balbina, Amazonas, Brazil. The offspring were transported to the Laboratory of Electronic Microscopy and Nanotechnology - LTMN, at the National Institute for Research in the Amazon - INPA, where they were euthanized with an overdose of thiopental (93 mg/kg) administered by intravenous injection.

After confirmation of death, the offspring were dissected by removing the plastron, and the mesonephros, gonads and ducts were removed and fixed in 10% formaldehyde solution with pH 7.2 and 0.1 M phosphate buffer for 12 h for microscopy light and immunohistochemistry. As there is no sexual dimorphism in the offspring of *P. expansa* the collections were performed randomly, we did not have the same proportion of males and females, allowing us to analyze males at one month (n = 1), three months (n = 1) and 12 months (n=1), and females at two months (n=1) and four months (n=1) posthatching.

### Immunohistochemistry of aromatase and AR, ER1 and ER2 receptors

After fixation, the samples were subjected to histological processing for inclusion in paraplast (Paraplast Plus^®,^ Sigma‒Aldrich), and subsequent histological sections of 4 μm were obtained via a microtome (RM2245), as previously described by Magalhães et al. (2023). The sections were placed on silanized slides (Perfect®, Perfecta LTDA, São Paulo, Brazil). The slides were subsequently deparaffinized in an oven at 54°C for 30 minutes, followed by immersion in xylene (2x) for 10 minutes each. For dehydration, 100% ethanol (3x), 95%, 80% and 70% ethanol solutions were immersed for five minutes each, and finally, the samples remained in distilled water for the same amount of time.

Antigenic retrieval was performed by immersing the slides in sodium citrate solution (pH 6.0), placing them in a conventional microwave oven at maximum power (750 W) until boiling, and maintaining them for 15 minutes according to the manufacturer’s instructions (Thermo Fisher Scientific, Massachusetts, USA). After cooling, the samples were washed in PBS-T solution (phosphate-buffered saline with Tween-20) under light agitation for 5 minutes.

To block endogenous peroxidase activity, the slides were immersed in 1% hydrogen peroxide solution for 10 minutes. After that, two washes were performed in PBS-T under light agitation, each lasting 5 minutes.

To block nonspecific binding, 300 μL of blocking solution (UltraCruz© Blocking reagent – SC 516214, Santa Cruz Biotechnology, Texas, USA) was added to the sections until they were covered. The samples were subsequently stored in a humid chamber for 30 minutes at room temperature. This was followed by two washes in PBS-T under light agitation for 5 minutes each.

For the incubation of the primary antibodies, 30 to 50 µL of the solution of the primary antibodies diluted in PBS were instilled in each histological section at the concentrations described in Table 1. The slides were incubated for 12 h (overnight) in a humid chamber at 4°C. The following day, the slides were washed 3 times with PBS under light agitation for 5 minutes each. All the sections were subsequently incubated with a solution containing the secondary antibody conjugated to HRP (goat anti-rabbit IgG H&L, G-21234; Thermo Fisher Scientific, Massachusetts, USA) at a concentration of 2:500; 30-50 µL of the secondary antibody was added to the sections, which were incubated in a humid chamber for 60 minutes at room temperature and then washed 3 times in PBS under light agitation for 5 minutes each.

**Table 1:** Specifications of the antibodies used and their respective dilutions.

**Code**	**Name**	**Concentration**	**Manufacturer**
PA1110	Rabbit AntiAndrogen Receptor Polyclonal Antibody	1:50	Invitrogen - Thermo Fisher Scientific - BR
PA121398	Rabbit Anti-Aromatase Polyclonal Antibody	1:100	Invitrogen - Thermo Fisher Scientific - BR
PA1311	Rabbit Anti-Estrogen Receptor Beta Polyclonal Antibody	1:250	Invitrogen - Thermo Fisher Scientific - BR
PA516476	Rabbit Anti-Estrogen Receptor Alpha Polyclonal Antibody	1:50	Invitrogen - Thermo Fisher Scientific - BR

In the dark, one drop of the chromogenic agent diaminobenzidine (DAB; Thermo Fisher Scientific, Massachusetts, USA) was instilled into each section for a period of 2 -7 minutes. The slides were subsequently washed 3 times in PBS under light agitation for 5 minutes each. Counterstaining was performed with Harris hematoxylin for 10 seconds, after which the samples were washed in water, dipped twice in absolute alcohol and once in xylene, and then mounted with Entellan®.

Positive reactions were recognized by the reddish-brown color, whereas negative reactions were not labeled. Negative controls involved the omission of incubation with the primary antibodies in the procedure. The validation of antibody specificity and immunolabeling patterns had been previously established in earlier studies from our group (Macedo et al., 2022), which served as positive controls in the present analysis. The sections were photomicrographed via a light microscope (Zeiss - axioplan2) coupled to an axiocamMRc camera to capture images of the reaction.

### Intensity of the immunoreaction by structure

To quantify the intensity of the positive reactions of each structure via the methodology adapted from Otsuka et al. (2008) the slides were analyzed blindly by three independent observers according to the following criteria: absent (0), weak (1), moderate (2), strong (3) and very strong (4). The final grade of the structures for each phase was assigned on the basis of the median of the observers.

For this evaluation, six photomicrographs were randomly obtained from three histological sections of each organ evaluated, as described above. The sections were obtained following a semiserial pattern every 30 μm.

The data were statistically analyzed via the Kruskal‒Wallis test, followed by Dunn's post hoc test, and the results were presented as the median, followed by the maximum and minimum values. The significance level adopted was 0.05. All analyses were performed via GraphPad Prism software.

### Authorizations for research

The study was licensed under the Biodiversity Information and Authorization System (SISBIO) n° 73951-1 of the Chico Mendes Institute for Biodiversity Conservation (ICMBio)/Ministry of the Environment (MMA). The laboratory procedures involving animals are in accordance with the standards established by the National Council for the Control of Animal Experimentation (CONCEA) and were approved by the Ethics Committee on the Use of Animals (CEUA) of the Federal University of Amazonas, under registration number: n^o^ 082/2019.

## Results

### Mesonephros

**Figure 1:**
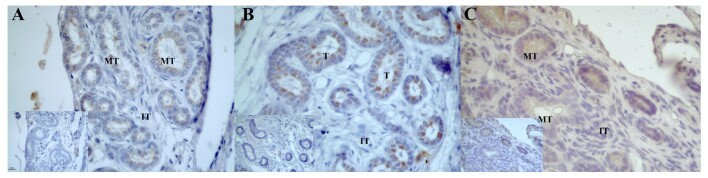
Photomicrograph of aromatase (AROM) immunoreaction in the degenerating mesonephros of post-hatching *P. expansa.* A – 1 month (male). B – 12 months (male) - mesonephros differentiating into the male reproductive duct. C – 2 months (female). **MT –** mesonephric tubules; **IT** – interstitial tissue; **T** – mesonephros tubule differentiating into epididymis.

Mesonephros were identified only in individuals up to 2 months posthatching. AROM it was observed in the apical region of the cells of the mesonephric tubules, where we detected weak to moderate expression in 1-month-old males (Figure 1A) and 12-month-old males in the mesonephros already in the process of differentiation to the epididymal duct (Figure 1B), and no significant differences were detected when the ages of the individuals were compared (Table 2). In females aged 2 months, a more diffuse reaction was observed in the parenchyma of the mesonephros (Figure 1C).

**Table 2:** Intensity [median (maximum - minimum)] of immunostaining of aromatase (AROM), estrogen receptors (ER1 and ER2), and androgen receptors (AR) in the mesonephros of male *Podocnemis expansa* during the first year of post-hatching life.

**Mesonephros – Male**												
	**AROM**			**AR**			**ER1**			**ER2**		
**Age (months)**	**1**	**3**	**12**	**1**	**3**	**12**	**1**	**3**	**12**	**1**	**3**	**12**
Mesonephric tubules	1.00 (0.00-1.00)	0.00 (0.00-1.00)	2.00 (0.00-3.00)	1.00 (1.00-1.00)	0.00 (0.00-1.00)	0.00 (0.00-0.00)	2.00a (2.00-3.00)	0.00b (0.00-0.00)	1.00^a^ (0.00-2.00)	1.00 (1.00-2.00)	0.00 (0.00-0.00)	1.00 (0.00-2.00)
Collecting ducts	0.00 (0.00-0.00)	0.00 (0.00-0.00)	0.00 (0.00-0.00)	0.00 (0.00-0.00)	0.00 (0.00-0.00)	0.00 (0.00-0.00)	0.00 (0.00-2.00)	0.00 (0.00-0.00)	0.00 (0.00-3.00)	0.00 (0.00-1.00)	0.00 (0.00-0.00)	0.00 (0.00-2.00)

^a-b^Indicates difference between months for the same marker (ER1), p<0.01.

**Figure 2:**
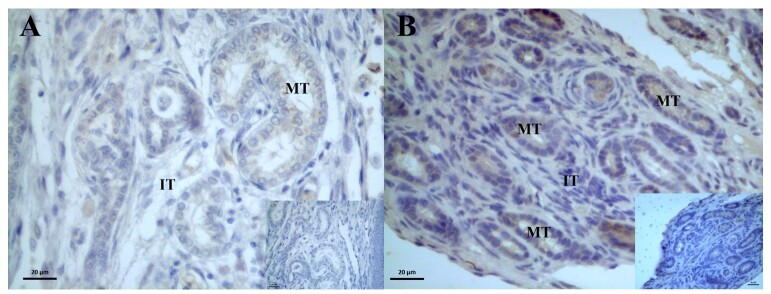
Photomicrograph of androgen receptor (AR) immunoreaction in the degenerating mesonephros of post-hatching *P. expansa*. A – 1 month (male). B – 2 months (female). **MT** – mesonephric tubules; **IT** – interstitial tissue.

The AR receptors immunoreaction was weakly expressed in the mesonephric tubules of 1-month-old males (Figure 2A), and in 2-month-old females, moderate to intense expression of AR receptors was detected in the distal tubules and mesonephric ducts, whereas weak expression was detected in the intertubular tissue (Figure 2B).

**Figure 3:**
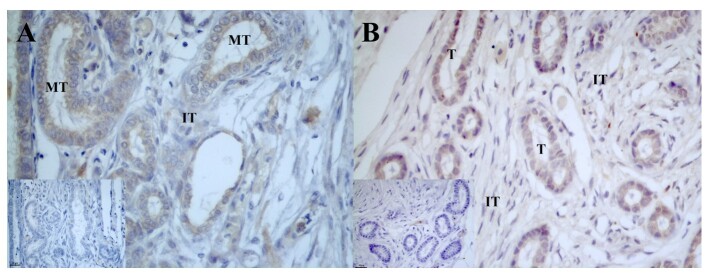
Photomicrograph of estradiol beta (ER2) receptor immunoreaction in the degenerating mesonephros of post-hatching *P. expansa*. A – 1 month (male). B – 12 months (male) - mesonephros differentiating into the reproductive duct. **MT** – mesonephric tubules; **IT** – interstitial tissue; **T** – mesonephros tubule differentiating into epididymis.

In the mesonephros of males aged 1 month, a moderate to strong immunoreaction of ER2 receptors was observed in the mesonephric tubules (Figure 3A), and in individuals aged 12 months, an immunoreaction was observed in the cells that compose the developing epididymal duct (Figure 3B). No significant differences were found in the intensity of the immunoreaction when the ages of the individuals were compared (Table 2).

**Figure 4:**
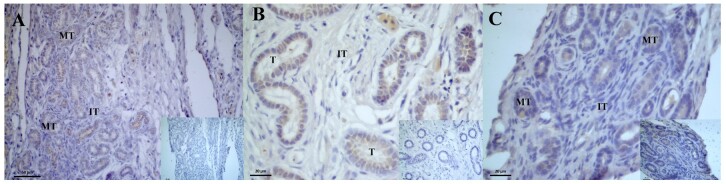
Photomicrograph of estradiol alpha (ER1) receptor immunoreaction in the degenerating mesonephros of post-hatching *P. expansa*. A – 1 month (male). B – 12 months (male) - mesonephros differentiating into the reproductive duct. C – 2 months (female). **MT** – mesonephric tubules; **IT** – interstitial tissue; **T** – mesonephros tubule differentiating into epididymis.

In all individuals analyzed, the immunolocalization of ER1 receptors was weak to moderate in the mesonephric and epididymal tubules (Figures 44B); statistically, we found a significant increase in the intensity of immunostaining in the mesonephric tubules when comparing month 1 to month 3 (Table 2). In the collecting ducts of a 2-month-old female (Figure 4C), the ER1 receptors were weakly expressed in the intertubular tissue. No significant differences in intensity were found when comparing the ages of the individuals (Table 3).

**Table 3:** Intensity [median (maximum - minimum)] of immunostaining of aromatase (AROM), estrogen receptors (ER1 and ER2), and androgen receptors (AR) in the mesonephros of female *Podocnemis expansa* during the first year of post-hatching life.

**Mesonephros - Female**								
	**AROM**		**AR**		**ER1**		**ER2**	
**Age (months)**	**2**	**4**	**2**	**4**	**2**	**4**	**2**	**4**
Mesonephric tubules	1.00	0.00	1.00	0.00	0.00	0.00	2.00*	0.00*
	(0.00-3.00)	(0.00-0.00)	(1.00-2.00)	(0.00-0.00)	(0.00-0.00)	(0.00-0.00)	(1.00-2.00)	(0.00-0.00)
Collecting ducts	0.00	0.00	0.00	0.00	0.00	0.00	0.00	0.00
	(0.00-1.00)	(0.00-0.00)	(0.00-3.00)	(0.00-0.00)	(0.00-0.00)	(0.00-0.00)	(0.00-1.00)	(0.00-0.00)

* Indicates difference between months for the same marker (ER2), p<0.01

### Testicles

**Figure 5:**
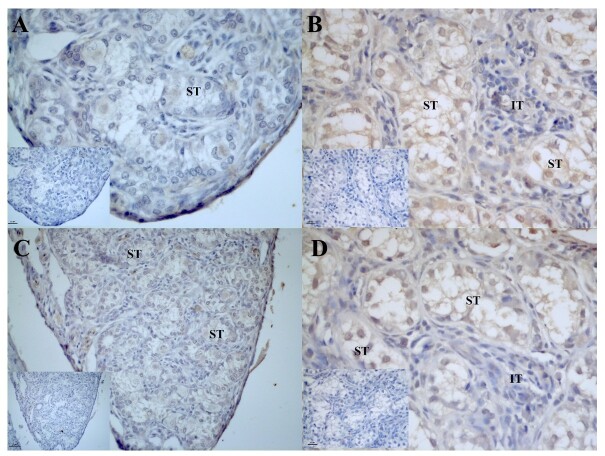
Photomicrograph of aromatase (AROM) enzyme immunoreaction and androgen receptor (AR) immunolocalization in the testicle of post-hatching *P. expansa*. A and B: Aromatase. C and D: Androgens. A – 1 month, B – 3 months, C – 1 month, D – 3 months. **ST** – seminiferous tubule; **IT** – interstitial tissue.

In individuals aged 1 month, AROM immunostaining was weakly expressed in the germ cells located within the seminiferous tubules and in some cells located in the interstitial tissue (Figure 5A). In 3-month-old individuals, the reaction was moderate, with AROM being identified in all cells that compose the seminiferous tubules and weakly or absent in cells of the interstitial tissue (Figure 5B). However, no significant difference was found between individuals of different ages (Table 4).

**Table 4:** Intensity [median (maximum - minimum)] of immunostaining of aromatase (AROM), estrogen receptors (ER1 and ER2), and androgen receptors (AR) in the testis of *Podocnemis expansa* during the first year of post-hatching life.

**Testicle**												
	**AROM**			**AR**			**ER1**			**ER2**		
**Age (months)**	**1**	**3**	**12**	**1**	**3**	**12**	**1**	**3**	**12**	**1**	**3**	**12**
Seminiferous tubules	1.00 (1.00-1.00)	2.00 (1.00-2.00)	0.00 (0.00-0.00)	1.00 (1.00-1.00)	2.00 (1.00-2.00)	0.00 (0.00-0.00)	1.00 (1.00-2.00)	2.00 (2.00-2.00)	1.00 (1.00-2.00)	2.00a^c^ (2.00-3.00)	2.00b (2.00-2.00)	0.00^ab^c(0.00-1.00)
Interstitial (endocrine) cell	0.00 (0.00-1.00)	1.00 (1.00-1.00)	0.00 (0.00-0.00)	2.00^ac^ (2.00-2.00)	0.00 (0.00-2.00)	0.00^ac^ (0.00-0.00)	3.00^ab^ (3.00-4.00)	0.00^ab^ (0.00-1.00)	1.00 (1.00-1.00)	1.00 (0.00-1.00)	2.00 (0.00-2.00)	0.00 (0.00-0.00)
Spermatogonia	1.00 (1.00-1.00)	2.00 (1.00-2.00)	0.00 (0.00-0.00)	1.00 (1.00-2.00)	2.00^bc^ (2.00-2.00)	0.00^bc^ (0.00-0.00)	2.00 (1.00-2.00)	2.00 (2.00-2.00)	2.00 (1.00-2.00)	2.00^ac^ (2.00-3.00)	2.00^bc^ (2.00-2.00)	0.00^ac^ (0.00-0.00)
Interstitial tissue	1.00 (1.00-1.00)	0.00 (0.00-1.00)	0.00 (0.00-0.00)	1.00^ac^ (1.00-2.00)	1.00 (0.00-2.00)	0.00^ac^ (0.00-0.00)	1.00 (0.00-1.00)	1.00 (0.00-1.00)	0.00 (0.00-1.00)	0.00 (0.00-0.00)	0.00 (0.00-0.00)	0.00 (0.00-0.00)

^a-c^Indicates difference between months for the same marker (AR) (ER1) (ER2), p<0.01

The AR receptors immunoreaction in the testis was observed in individuals at one, three and twelve months after hatching; however, this reaction was greater in individuals at three months of age. There was a weak AR nuclear immunoreaction in the cells that composed the seminiferous tubules in the first month, whereas in the third month, this reaction was moderate (Figure 55D). Data on intensity variation were significantly different in interstitial cells and tissue when individuals aged one and twelve months were compared (Table 4), and there was a significant difference in the germ cells of individuals aged three and twelve months (Table 4).

**Figure 6:**
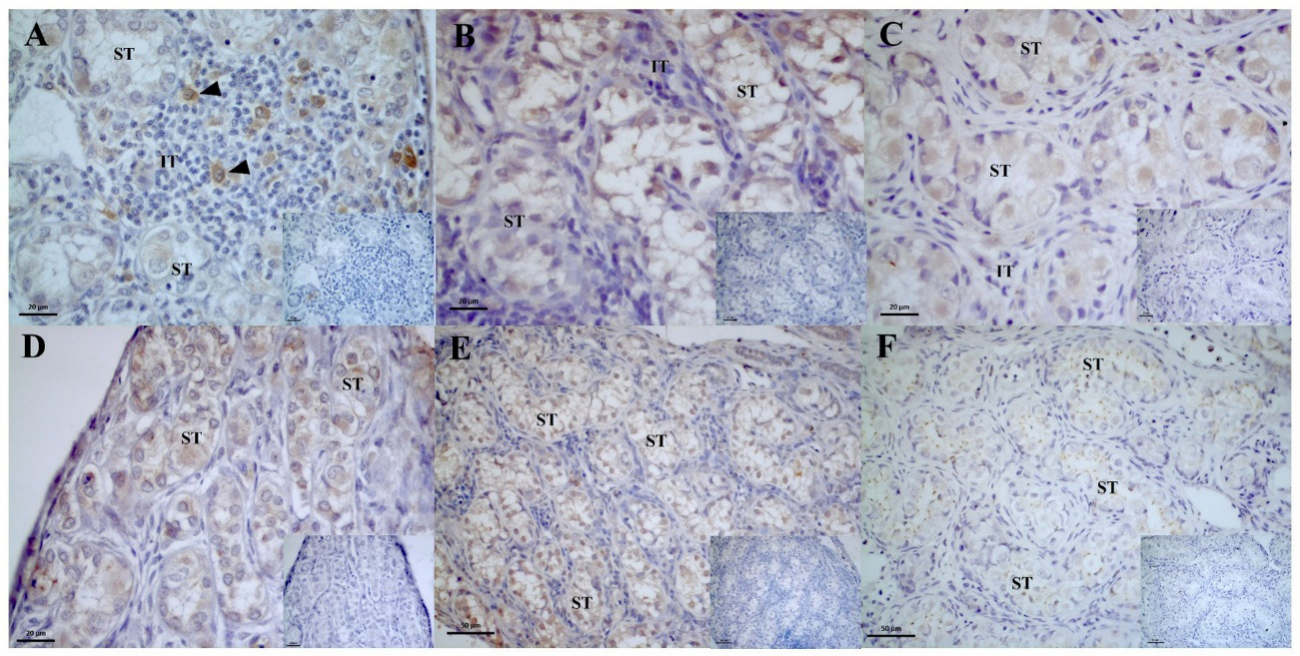
Photomicrograph of estradiol beta (ER2) and alpha (ER1) receptor immunoreaction in the testicle of post-hatching *P. expansa*. A – C: estradiol beta. D – F: estradiol alpha. A – 1 month. B – 3 months. C – 12 months. D – 1 month. E – 3 months. F – 12 months. **ST** – seminiferous tubule; **IT** – interstitial tissue; ►- endocrine cells.

An analysis of the immunolocalization of ER2 receptors revealed that in 1-month-old individuals, the spermatogonia presented weak to moderate expression, and in the interstitial tissue endocrine cells were identified in the interstitial region with strong expression of ER2 receptors (Figure 6A). We identified a significant difference when comparing individuals aged one and twelve months and between individuals aged three and twelve months, and no significant differences were found between individuals aged one and three months for ER2 immunostaining in spermatogonia (Table 4). In individuals aged 3 and 12 months, the cells that were part of the seminiferous tubules presented moderate (Figure 6B) to weak (Figure 6C) immunostaining for ER2 receptors, and in the interstitial tissue, immunostaining was identified at low intensity.

ER1 receptor was immunolocalized in the cells that compose the seminiferous tubules and was weak to moderate in individuals aged 1 month (Figure 6D), moderate in 3-month-old subjects (Figure 6E) and weak in 12-month-old subjects (Figure 6F). In the interstitial tissue, regardless of age, immunostaining was not detected, but we detected a significant difference in the immunoreaction of interstitial cells when individuals aged one and three months were compared (Table 4).

### Ovary

**Figure 7:**
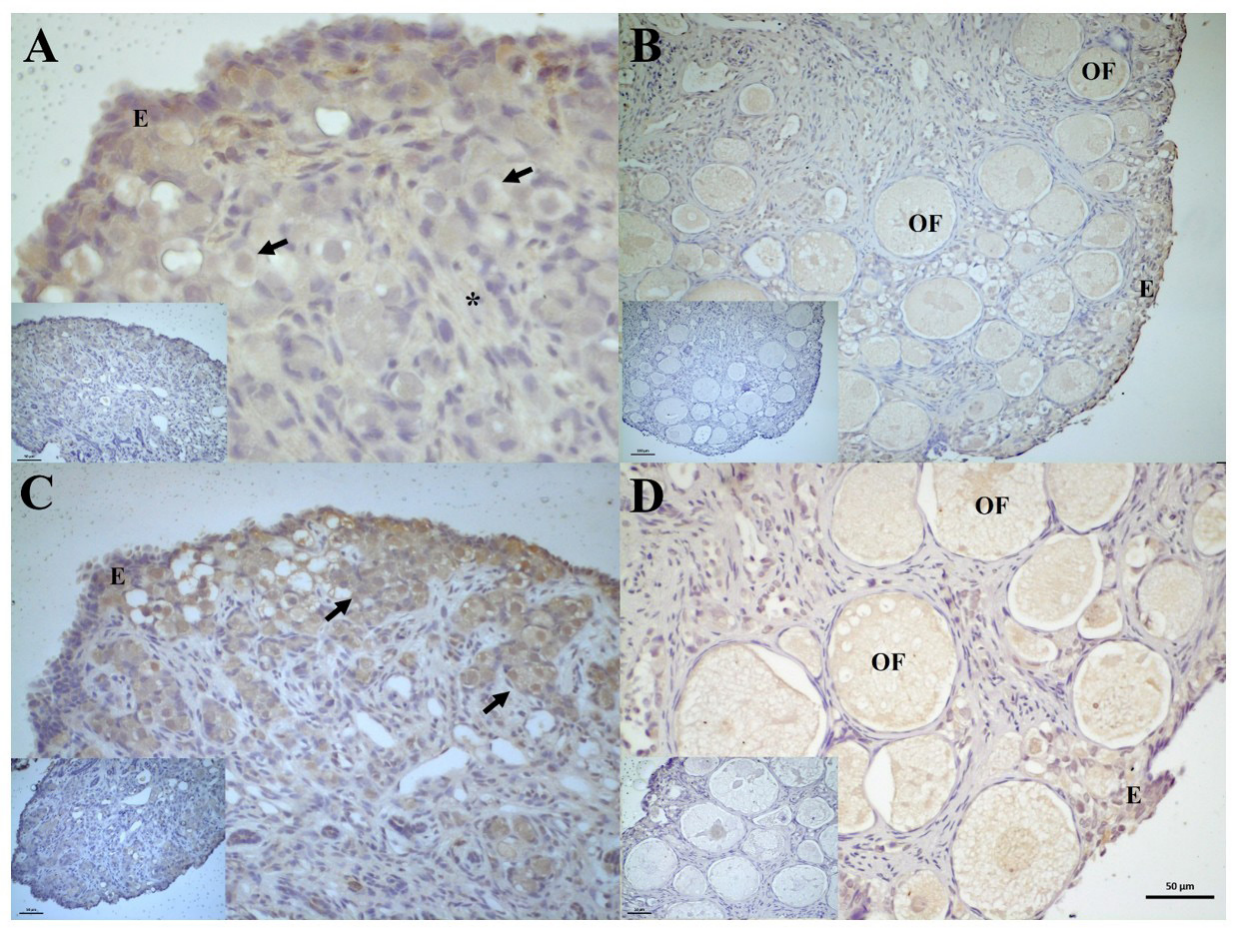
Photomicrograph of aromatase (AROM) enzyme immunoreaction and androgens receptors (AR) in the ovary of post-hatching *P. expansa*. A and B: Aromatase. C and D: Androgens. A – 2 months, B – 4 months. **E** – epithelium; Oogonia; * - ovarian stroma; **OF** – ovarian follicles.

There was moderate immunostaining of AROM in the germinal epithelium and oogonia and moderate to weak immunostaining in the stroma of the ovaries of two-month-old individuals (Figure 7A). In the ovaries of four-month-old individuals, a weak to moderate reaction was observed in the germinal epithelium, a weak reaction was detected in the ovarian follicles and ovarian stroma, and no reactions were detected in the follicular cells (Figure 7B).

The AR receptors immunoreaction was intense in the germinal epithelium and in the oogonia located in the ovarian cortex of the ovaries of two-month-old individuals (Figure 7C). In the ovaries of 4-month-old individuals, weak expressions were detected in the germinal epithelium, and weak to moderate immunostaining was detected in the oogonia and ovarian follicles (Figure 7D).

**Figure 8:**
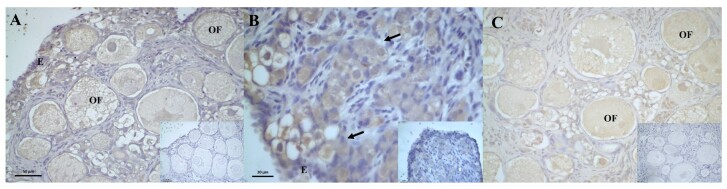
Photomicrograph of estradiol beta (ER2) and estradiol alpha (ER1) receptor immunoreaction in the ovary of post-hatching *P. expansa*. A: estradiol beta; B and C: estradiol alpha. A - 4 months, B – 2 months, C – 4 months. **E** – epithelium; oogonia; * - ovarian stroma; **OF** – ovarian follicles.

The ER2 receptor in the 4th month ovary showed weak to moderate immunoreactivity in the germinal epithelium, oogonia and ovarian follicles (Figure 8A). A significant variation in the immunoreaction of the oogonia was identified when the individuals aged two and four months were compared (Table 5). In the interstitial tissue, no cell immunostaining was observed. The ER1 receptor in the 2-month-old ovary showed weak immunostaining in the germinal epithelium and strong to intense immunostaining in the oogonia (Figure 8B). In the ovaries of four-month-old individuals, weak ER1 expressions were detected in the follicles and oogonia, and in the germinal epithelium (Figure 8C).

**Table 5:** Intensity [median (maximum - minimum)] of immunostaining of aromatase (AROM), estrogen receptors (ER1 and ER2), and androgen receptors (AR) in the ovary of *Podocnemis expansa* during the first year of post-hatching life.

**Ovaries**								
	**AROM**		**AR**		**ER1**		**ER2**	
**Age (months)**	**2**	**4**	**2**	**4**	**2**	**4**	**2**	**4**
Oogonia	2.00	1.00	3.00	1.00	2.00	0.00	3.00*	1.00^*^
	(2.00-2.00)	(1.00-2.00)	(3.00-4.00)	(1.00-2.00)	(1.00-2.00)	(0.00-0.00)	(3.00-4.00)	(0.00-2.00)
Ovarian Follicles	0.00	1.00	0.00	1.00	0.00^*^	1.00^*^	0.00	1.00
	(0.00-2.00)	(1.00-1.00)	(0.00-2.00)	(1.00-2.00)	(0.00-0.00)	(1.00-2.00)	(0.00-1.00)	(0.00-1.00)
Ovarian Stroma	1.00	1.00	1.00	1.00	0.00	0.00	0.00	1.00
	(0.00-2.00)	(1.00-1.00)	(1.00-1.00)	(1.00-1.00)	(0.00-0.00)	(0.00-1.00)	(0.00-1.00)	(0.00-1.00)

* Indicates difference between months for the same marker (ER1 e ER2), p<0.01

## Discussion

The present study is pioneering in investigating the immunolocalization of androgen (AR), estrogen (ER1 and ER2) and aromatase (AROM) receptors in the gonads and mesonephros of Amazonian turtles (*Podocnemis expansa*) from hatching to the first year of life. The variation in the spatiotemporal distribution of hormone receptors observed in the gonads and mesonephros of males and females during the first year of life suggests that these hormones also play important roles in the endocrine control of the posthatch development of the urogenital system in these turtles.

Mesonephros, in addition to their excretory role during embryonic development, have already been described as a possible extragonadal source of estrogens due to the presence of the aromatase enzyme, which plays an important role in the development and gonadal differentiation of Amazon turtles (Magalhães, 2017; Macêdo et al., 2022). In the present study, an immunoreaction for AROM and steroid sex hormone receptors was identified in the mesonephros up to two months of age, suggesting that this organ may continue to synthesize estrogens and influence gonadal maturation after hatching in this species. In addition, in males, mesonephros begin to differentiate into the epididymis after hatching, and the immunoreaction of these receptors indicates the influence of these hormones on this process (Chevrel et al., 1965; Kardong, 2010). In females, moderate AROM receptors immunoreactivity occurs in the mesonephros and diffuses both in the tubules and in the interstitial tissue up to two months of age, suggesting that this organ may still be an important source of estrogen for ovarian maturation.

Androgens are essential hormones for sexual development and maturation and are required in males for spermatogenesis and the development of the reproductive tract and, in females, for the process of folliculogenesis (Yeh et al., 2002; De Gendt et al., 2004; Matsumoto et al., 2008). The immunolocalization of AR receptors in *P. expansa* after hatching at different ages in males and females reinforces the importance of these hormones in testicular and ovarian maturation in this juvenile phase. Although we did not find a progressive increase in the intensity of AR receptor immunolocalization throughout posthatch development, it is important to consider that hormone levels tend to increase only near or during the reproductive period (Jones and Swain, 1996; Jones et al., 1997; Alcock, 2011). As the individuals analyzed in this study were still in the immature phase, the absence of this increase can be explained by the fact that sexual maturity in *P. expansa,* as in other chelonian species, usually begins after seven years of age and is correlated mainly with body size (Cagle, 1950; Gibbons, 1968; Ernst, 1971).

In zebrafish, the loss of AR function results in defects in oocyte maturation and in the structural organization of the testis (Crowder et al., 2018). Similar findings have been described in frogs (*Xenopus laevis*), where androgens regulate oocyte maturation (Lutz et al., 2001), and in female *Alligator mississippiensis*, during postnatal development, testosterone reportedly plays an important role in the development of ovarian follicles (Moore et al., 2010). Our findings revealed that AR receptors immunolocalization was more intense in 2-month-old females than in 4-month-old females, suggesting that this hormone may play a critical role in the initial maturation of follicles and oocytes in *P. expansa*. In mice, androgen-deficient females presented an increase in the number of atretic follicles and a reduction in the number of corpora lutea, resulting in infertility (Matsumoto et al., 2008), which reinforces the importance of androgens in posthatch ovarian development in turtles.

Estrogens act mainly through their specific receptors, and the expression levels of these receptors may reflect the sensitivity of tissues to these hormones. Estrogens are involved in gonadal differentiation, reproductive tract maturation and reproductive behavior (McLachlan, 2001; Iguchi et al., 2006). Our findings revealed an immunoreaction for ER1 and ER2 receptors in the ovarian follicles, germinal epithelium and oogonia, suggesting an active role of these hormones in follicular growth and maturation. Studies in *Xenopus laevis* have shown that estrogen synthesis occurs significantly in oocytes, a common feature among vitellogenic vertebrates (Gohin et al., 2011).

The vertebrate testis can be both a source and a target for estrogenic hormones (O'Donnell et al., 2001; Akingbemi, 2005). These estrogens can act in a paracrine manner in the testis or affect distant tissues. The location of estrogen receptors in testicular tissue varies between species and depends on the developmental stage (Abney, 1999; O'Donnell et al., 2001; Hess and Carnes, 2004). In our findings, in addition to the immunolocalization of ER1 and ER2 receptors in spermatogonia, we identified their presence in interstitial cells, probably Leydig cells, with a predominance of ER2. This reinforces the relevance of these hormones in testicular physiology, with a possible influence on spermatogenesis and androgen synthesis, in this process of initial maturation of the gonads after hatching.

Despite the very small and sex-unbalanced sample, given that hatchlings were collected randomly and lack external sexual dimorphism, our semiquantitative, blinded analyses across multiple sections/fields yielded consistent interpretations and support meaningful discussions of the underlying biological processes. These results should be viewed as hypothesis-generating rather than definitive. Future studies should incorporate finer age bins, include quantitative protein/RNA assays (e.g., Western blot/RT-qPCR for ER1/ER2/AR/AROM), and functional readouts (e.g., hormone levels and ex vivo receptor antagonism) to test the mechanistic links suggested by the present immunolocalization. To our knowledge, this is the first study to demonstrate the immunolocalization of aromatase and sex steroid receptors *in Podocnemis expansa* during posthatching development. Although direct molecular evidence in this species is still lacking, studies in other vertebrates, such as fish, birds, and mammals, have shown that transcriptional, hormonal, and epigenetic mechanisms tightly control the spatial and temporal expression of aromatase and steroid receptors during gonadal differentiation (Guiguen et al., 2010; Nakabayashi et al., 1998; Hinfray et al., 2017; Driscoll et al., 2020; Wu et al., 2020; Yan et al., 2021). Moreover, evidence from neuroendocrine and comparative studies indicates that environmental cues and feedback from gonadal hormones can modulate these pathways, influencing sexual differentiation and plasticity across vertebrate lineages (Brock et al., 2015; Tenugu et al., 2021; Rosati et al., 2021; Gegenhuber et al., 2022). These integrated findings provide a broader framework to interpret the steroidogenic dynamics observed here in *P. expansa* and highlight potential conserved regulatory mechanisms among species with temperature-dependent sex determination.

Our findings are highly important for understanding the reproductive physiology of *Podocnemis expansa*. By demonstrating the presence of and variation in the expression of aromatase, androgen and estrogen receptors throughout the first year of life, we provide a basis for future research on the endocrine control of reproduction in this species during this growth phase. In addition, these results may have significant implications for conservation and reproductive management programs, helping to formulate strategies based on the reproductive biology of the species, which are fundamental for the preservation of *P. expansa* in natural and controlled environments.

## Conclusion

This study investigated the immunolocalization of androgen, estrogen and aromatase receptors in the gonads and mesonephros of Amazonian turtles (*Podocnemis expansa*) during the first year of life. The immunolabeling patterns of these molecules varied according to tissue distribution, age and sex, indicating the influence of sex steroids on the maturation of these organs after hatching until the first year of life. These findings advance the understanding of early reproductive endocrinology in chelonians and provide a basis for future studies on the endocrine control of reproduction in this species. They may also support conservation and reproductive management programs grounded in reproductive biology. Furthermore, this knowledge can be extended to other turtle species with temperature-dependent sex determination (TSD), contributing to their preservation Although based on a limited and unbalanced sample, the results establish an important reference framework for subsequent quantitative and functional analyses.

## Data Availability

Research data is only available upon request.
